# Choroidal thickness as a biomarker of systemic inflammation in patients with polymyalgia rheumatica

**DOI:** 10.3389/fmed.2025.1689327

**Published:** 2025-10-17

**Authors:** Laura Trives-Folguera, Santiago Muñoz-Fernández, María del Mar Esteban-Ortega, Raquel Coca-Serrano, Tatiana Cobo-Ibañez, Cristina Vergara-Dangond, Liz Romero-Bogado, Isabel de la Cámara-Fernández, Patricia Richi, María Beatriz Paredes, Ana Esteban-Vazquez, Ana Valeria Acosta, Gabriela Cueva Najera, Marco Algarra San José, Jorge Juan González-Martín, Karen N. Franco Gomez, Patricia Bogas Schay, Tamara Shukair Harb, Israel Thuissard-Vasallo, Martina Steiner

**Affiliations:** ^1^Department of Rheumatology, Hospital Universitario Infanta Sofía, Madrid, Spain; ^2^Department of Rheumatology, Hospital Universitario HM Sanchinarro, Madrid, Spain; ^3^Faculty of Medicine, Health and Sports, Department of Medicine, Universidad Europea de Madrid, Madrid, Spain; ^4^FIIB HUIS-HUHEN, Madrid, Spain; ^5^Department of Ophthalmology, Hospital Universitario Infanta Sofía, Madrid, Spain; ^6^Department of Ophthalmology, Hospital Universitario HM Sanchinarro, Madrid, Spain

**Keywords:** choroidal thickness, polymyalgia rheumatica, inflammatory activity, biomarker, optical coherence tomography

## Abstract

**Objective:**

Choroidal thickness (CT) varies with systemic inflammatory activity in diseases such as spondyloarthritis, suggesting its potential role as a biomarker. This study aimed to evaluate changes in CT in patients recently diagnosed with Polymyalgia Rheumatica (PMR) who are undergoing corticosteroid therapy, over a six-month follow-up period.

**Methods:**

It is a prospective, observational, longitudinal pilot study including 20 patients with recent PMR diagnosis from two centres. All participants met PMR classification criteria. Participants underwent three visits: at diagnosis (baseline), at 3 and 6 months after starting corticosteroids. Each visit included physical examination, musculoskeletal ultrasound (MSK US) of shoulders and hips, blood tests including C-reactive protein (CRP) and erythrosedimentation rate (ESR) and CT measurement by optical coherence tomography (OCT). Disease activity was assessed using the PMR Activity Score (PMR-AS) and its imputed version.

**Results:**

Mean baseline CT was 242.10 ± 79.05 μm. Choroidal thickness decreased significantly after 3 months (229.85 ± 79.01 μm, *p* = 0.017) and after 6 months (220.37 ± 75.96 μm, *p* = 0.014) of corticosteroid treatment. We found a significant decrease in all laboratory and clinical parameters. Concordance between CT, CRP, and PMR-AS was 95%. Rotator cuff pathology does not appear to influence on evolution of MSK US bicipital tenosynovitis inflammatory findings neither in pain.

**Conclusion:**

We found that CT was high in patients with recent diagnosis of PMR and decreases significantly after 3 and 6 months of corticosteroid therapy. There is a 95% of concordance between CT and CRP as well as between CT and PMR activity scores. Our findings suggest that CT is useful as a noninvasive, imaging-based biomarker of systemic inflammation in patients with PMR. More studies are needed to confirm these preliminary results.

## 1 Introduction

Polymyalgia rheumatica (PMR) is an inflammatory disorder characterized by bilateral moderate-to-severe pain and stiffness affecting the neck, shoulders, and hips (1). It is the most common inflammatory disease affecting individuals aged >50 years, mainly women. The incidence of PMR increases progressively with age and peaks between 70 and 79 years (2). The cardinal feature is new and acute-onset proximal girdle pain associated with stiffness for >45 min (3). Although the etiology of the disease is unknown, the contribution of environmental and genetic factors that cause inflammation of the affected joints has been suggested. In patients with PMR, laboratory tests show increased levels of acute phase reactants (APR), including C-reactive protein (CRP), and erythrocyte sedimentation rate (ESR), which are essential criteria for diagnosis (4, 5). The production of these reactants is mediated by IL-6, which is considered the key proinflammatory interleukin and, consequently, the central driver of systemic inflammation in PMR. This production appears to involve both an increase in immunoblasts in peripheral blood and a decrease in regulatory T cells, as well as direct activity within inflamed tissues, where an expansion of memory T cells has been observed (6).

Corticosteroids are the cornerstone of PMR treatment. Most patients exhibit rapid and substantial improvement within few days of treatment initiation. However, up to 50% of patients become corticosteroid-dependent, resulting in prolonged exposure and an increased risk of adverse effects. In such cases, treatment with IL-6 inhibitors is currently considered (7, 8).

Leeb et al. established the response criteria to treatment in patients with PMR, which include five markers: ESR or CRP level, pain measured using a visual analog scale (VAS), physician’s global assessment (PGA), duration of morning stiffness (MST) in minutes, and ability to elevate the upper limbs (EUL). The last marker is evaluated using a scale from 0 to 3. Based on these markers, the following formula has been developed to calculate the PMR disease activity score (PMR-AS) (9, 10).

PMR-AS = CRP (mg/dL) + VAS pain (0–10) + PGA (0–10) + (MST [min] x 0.1) + EUL (3–0).

Considering that patients with PMR are typically over 50 years old, and that both the VAS pain score and the EUL score may be influenced by coexisting mechanical and degenerative conditions, determining the exact origin of pain is often challenging. Furthermore, although increased acute phase reactant (APR) levels are useful for assessing PMR activity, they may not indicate disease relapse in asymptomatic patients. Moreover, in patients treated with IL-6 inhibitors, APRs (such as CRP and ESR) are suppressed and therefore cannot be used to monitor disease activity (11).

In autoimmune diseases, the vascular system is frequently affected, either through direct vessel inflammation or through accelerated atherosclerotic and thrombotic processes. Immune activation, including autoantibody production and cellular immune responses, can trigger vascular injury associated with intimal hyperplasia in small arterioles, leading to luminal narrowing and subsequent tissue hypoxia (12, 13). The choroid is the tissue in the human body with the highest blood flow per unit weight, making it susceptible to inflammation in multisystemic diseases. Previous studies have proposed and evaluated choroidal thickness (CT) measurement by optical coherence tomography (OCT) as a potential novel biomarker for systemic diseases (14). While many autoimmune diseases can cause specific ocular involvement, previous studies have reported CT changes associated with systemic inflammatory activity in patients without direct eye involvement. In systemic lupus erythematosus (SLE), CT appears to vary according to the degree of inflammation and disease progression. A thicker CT may indicate early, subclinical neuropathy and nephropathy, whereas in more advanced disease, CT tends to be thinner due to associated vascular damage (15, 16). A similar pattern is observed in patients with Behçet’s disease. During periods of acute inflammation, CT increases, whereas in patients with advanced disease, the choroid appears thinner due to chronic damage (17). Furthermore, as reported in several studies, choroidal involvement appears to occur independently of ocular involvement in Behçet’s disease (18). Vascular involvement in systemic sclerosis has been associated with choroidal thinning; however, these findings require confirmation through additional studies (19). There are controversial data regarding CT changes in patients with rheumatoid arthritis (RA). While some studies have reported thinner CT in RA patients, others have shown an increase in this ocular structure (20–22). The treatment regimens received could have influenced these controversial results, as no significant differences were reported in RA activity, disease duration, or radiologic damage. Previous studies have reported increased CT in patients with active spondyloarthritis, which decreased significantly following biological treatment (23). Han et al. demonstrated a significant increase in CT in patients with non-ocular sarcoidosis compared to healthy individuals, although no significant differences were observed in the choroidal vascularity index. The authors propose that choroidal thickening in these patients may reflect underlying subclinical inflammation (24). Based on these findings, CT could be a biomarker of systemic inflammation in patients with inflammatory diseases.

The subacute onset and rapid therapeutic response in PMR can be attributed to the reduction of IL-6, the key cytokine driving the inflammatory process. In other immune-mediated diseases, IL-6 does not play such a decisive role; rather, other interleukins are involved, which likely explains why the initial response to corticosteroids is less pronounced. Consequently, inhibition of the relevant cytokines effectively precludes alternative pathways that might otherwise influence disease activity or compromise the reliability of inflammatory markers such as CRP.

As mentioned above, corticosteroid-dependent patients require treatment with IL-6 inhibitors, which suppress the APR. However, some patients may experience clinical flares without corresponding increases in CRP or ESR. Therefore, there is a need for novel objective biomarkers to accurately assess systemic inflammatory activity in patients with PMR.

We hypothesized that in patients with PMR, CT decreases after corticosteroid therapy and is a biomarker of systemic inflammation. Therefore, this study aimed to evaluate CT changes in patients with recently diagnosed PMR at baseline and after 3 and 6 months of corticosteroid therapy. In addition, the study aimed to assess the correlation of CT and CRP level with disease activity indices and scales currently used in clinical settings at baseline and at 3 and 6 months of follow-up. Additionally, this study aimed to evaluate the correlation between changes in every item between visits (from baseline to 3 months; from baseline to 6 months, and from 3 months to 6 months). Finally, the concordance between CT and CRP level and disease activity scores was evaluated at baseline and after 3 and 6 months of treatment. To the best of our knowledge, this is a first study to evaluate CT changes in patients with PMR receiving corticosteroids.

## 2 Materials and methods

### 2.1 Study design

This observational, descriptive, prospective, longitudinal, multicentre pilot study included patients with recently diagnosed PMR.

### 2.2 Participants

Participants were recruited from two centres (University Hospital Infanta Sofía and University Hospital Hospitales Madrid (HM) Sanchinarro). All patients aged >18 years, who were recently diagnosed with PMR and met the PMR classification criteria were included (5). Patients with symptoms suggestive of PMR were diagnosed based on the clinical, laboratory, and musculoskeletal ultrasound (MSK US) findings. Patients with a history of inflammatory ocular disease, patients receiving corticosteroids for any other disease, and pregnant patients were excluded. All patients included in the study underwent a checklist of questions regarding potential symptoms suggestive of GCA (presence of fever, significant weight loss, headache, mandibular claudication), followed by a thorough physical examination including assessment of arterial pulses, particularly the temporal arteries. Patients presenting with symptoms suggestive of GCA were excluded from the study.

All investigators acted in accordance with the principles of the Declaration of Helsinki. Written informed consent was obtained from all participants. The institutional review board approved this study (Approval code, A06/24).

### 2.3 Examination protocol

At the baseline visit, a diagnosis of PMR was made and prednisone (10–15 mg/day per oral) was initiated. Additionally, sociodemographic and clinical data were collected.

Physical examination included the number of tender and swollen joints, EUL score, and MST. In addition, the VAS score for pain and PGA score were determined. Grades 0, 1, 2, and 3 EUL correspond to complete shoulder abduction (180°), abduction above shoulder level (≥90° and <180°), abduction below shoulder level (<90° and >0°), and inability to raise the arms, respectively.

The presence of biceps tenosynovitis (BT), subacromial and subdeltoid (SASD) bursitis, glenohumeral or axillary synovitis, trochanteric bursitis, and hip synovitis were evaluated using MSK US (Logiq E US, General Electric). A linear probe was used at a frequency of 8–12 Hz and adjusted according to the examination area. The same physician, with at least 5 years of experience, performed the examination during the follow-up. We evaluated BT since it was the most frequent MSK US finding seen in our patients at baseline.

Blood tests, including CRP levels and ESR, were performed in the laboratory unit. CRP levels were measured in mg/L and subsequently adjusted to mg/dL, to calculate the activity score. ESR values were obtained at all visits in only 12 (60%) patients owing to measurement errors.

**Figure 1:**
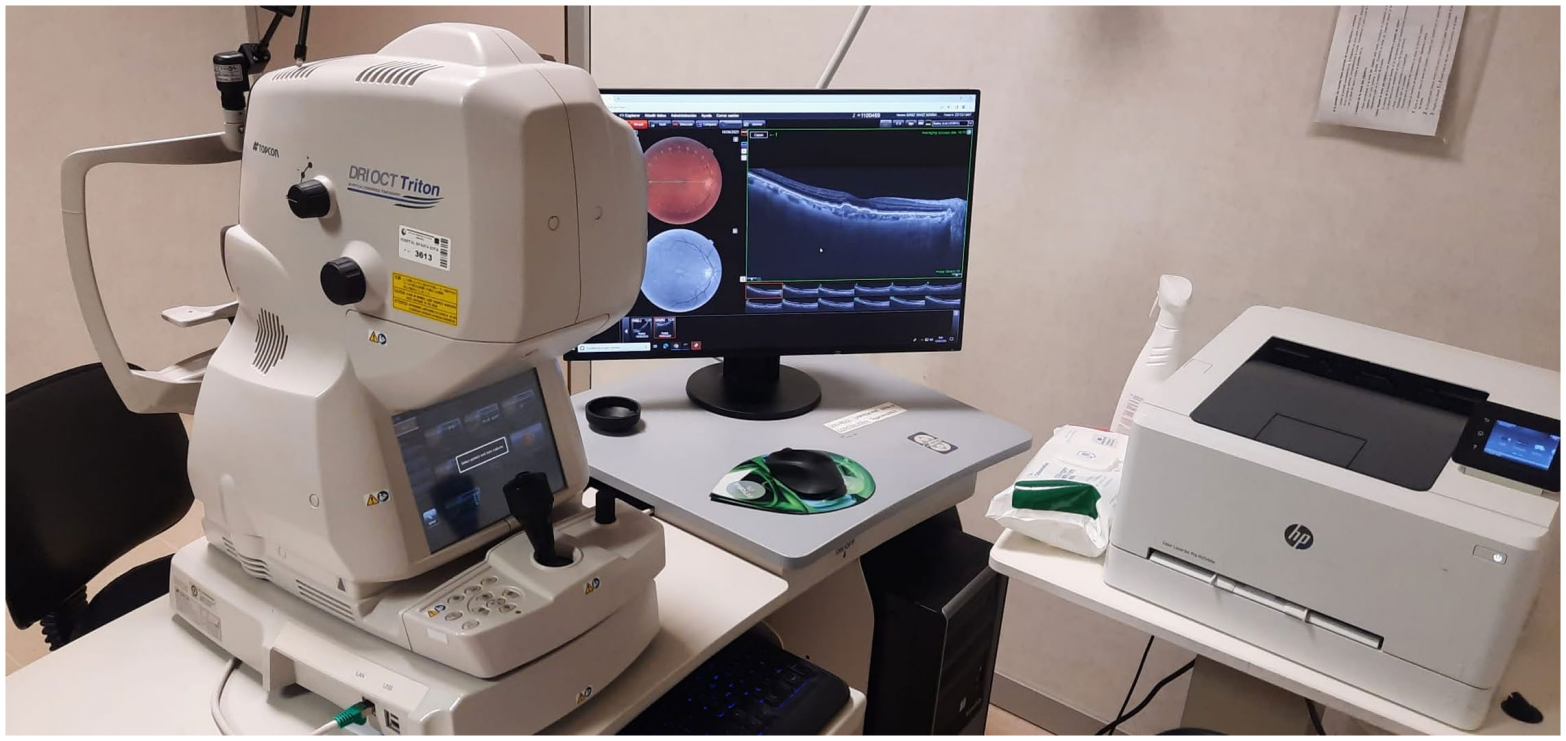
Topcon DRI-OCT triton swept-source optical coherence tomography (SS-OCT). picture provided by Dra. Esteban-Ortega, ophthalmology service.

**Figure 2:**
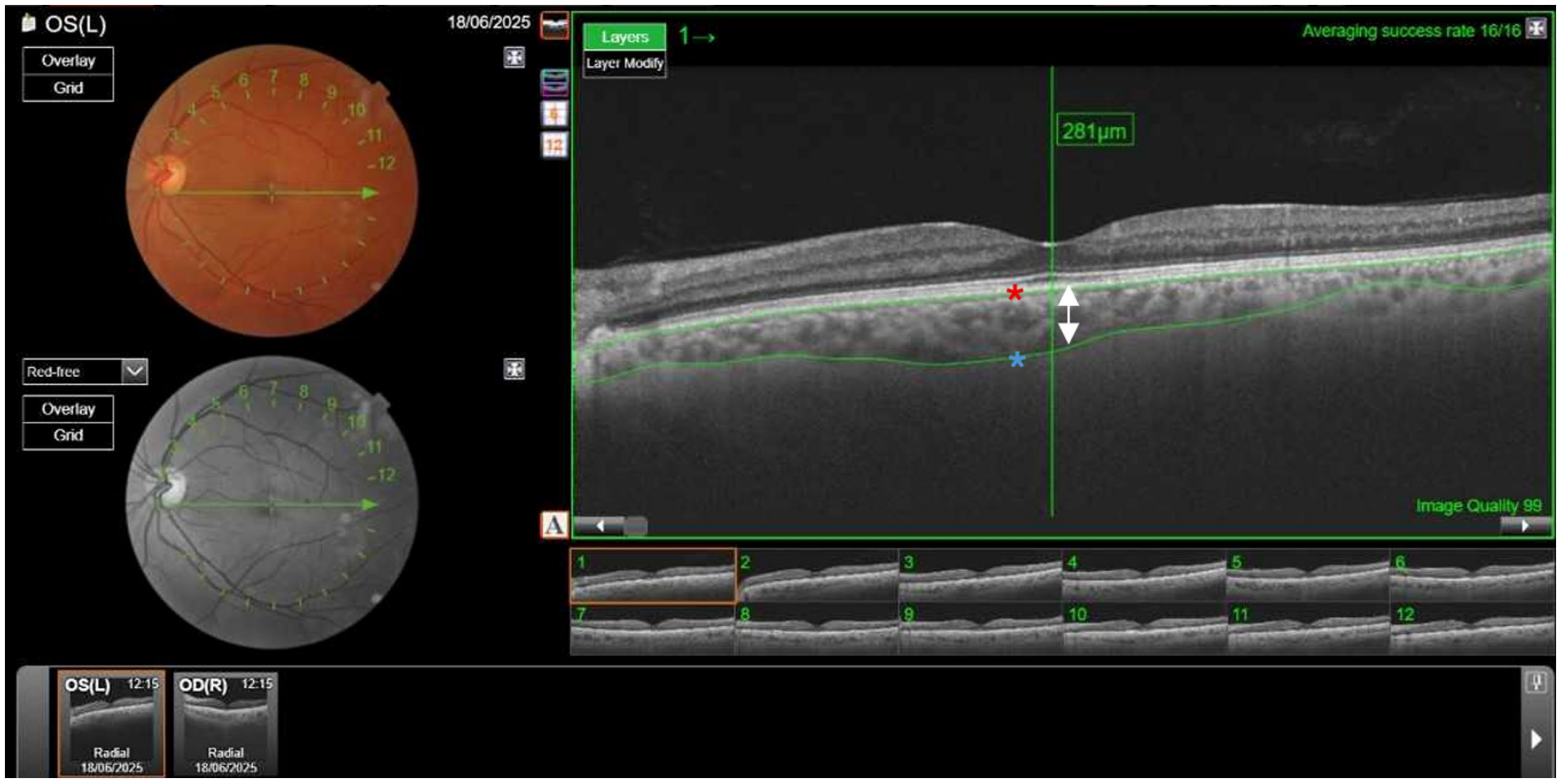
Measurement of choroidal thickness (CT) using Topcon DRI-OCT Triton Swept-Source-Optical coherence tomography (SS-OCT). Subfoveal CT was defined as the distance from the outer reflective line of the retinal pigment epithelium (red asterisk) to the inner limit of the sclera (blue asterisk). The arrow shows the thickness of the choroid layer, in this case, 281μm. (Picture provided by Dra. Esteban-Ortega, Ophthalmology service).

CT was measured using swept-source OCT (Topcon DRI-OCT Triton) (Figure 1). OCT is a non-invasive diagnostic technique that does not use ionizing radiation, is easy to perform, and does not require the use of mydriatic drugs. Swept-source OCT uses light waves with longer wavelengths, allowing greater penetration. The main advantage is an automatic CT measurement. Subfoveal CT was defined as the distance from the outer reflective line of the retinal pigment epithelium to the inner limit of the sclera—choroid junction indicated by a hyperfluorescent surface (Figure 2). CT measurements were obtained for both eyes of all participants. Based on the assumption of correlation between the CT in both eyes, the larger CT value for each patient was used for analysis (25, 26). A common statistical issue in precision studies is whether 1 or 2 eyes of patients should be used. The problem arises because of the greater correlation between the 2 eyes of an individual compared with the correlation between 2 individuals. Exceptions can occur in cases of asymmetric eye disease. According to several studies there are two options. The first option is to use only 1 eye of the individual. The second option is to use both eyes but control for the non-independence of measurements (27). Kim MS et al. (28) found no statistically significant differences in CT between the right and left eye in all corresponding areas. However, the nasal peripapillary and peripheral areas had relatively low correlation coefficients, compared to the macular areas. In our study a subfofeal CT, the regions with no anatomic variations between individuals.

All images were captured by a trained optometrist. An expert ophthalmologist verified the accuracy of CT measurement.

At the 3- and 6-month follow-ups, physical and MSK US examination, blood tests, and OCT were performed, and the prednisone dose was recorded.

PMR-AS was calculated at every visit. Values ≤1.5 points indicated remission whereas, values <7, 7–17, and >17, indicated low, moderate, and high disease activity, respectively (9). In addition to the PMR-AS, an imputed PMR-AS (PMR-ASimp), in which CRP was replaced by a value imputed based on the other score items, was calculated at every visit using the formula (29).


1.12(clin‐PMR‐AS)+0.26

where clin-PMR-AS is the sum of MST × 0.1, EUL, VAS score for pain, and PGA.

### 2.4 Sample size

Based on clinical experience, we hypothesized that in patients with PMR, improvement in inflammatory activity after 6 months of corticosteroid therapy would markedly reduce CT. However, we were unable to find scientific publications that indicate the magnitude of decrease that can support this hypothesis and allow us to calculate the required minimum sample size. Therefore, this study was proposed as a pilot study. All information was entered into a specific database designed for the study.

### 2.5 Statistical analysis

Descriptive statistics were used to summarize the study variables. For qualitative variables, absolute and relative frequencies are reported. Quantitative variables are expressed as mean ± standard deviation or median and interquartile range (p25–p75), based on the normality of distribution assessed using the Shapiro–Wilk test.

Clinical parameters were compared between the three timepoints (baseline, 3 months, and 6 months) using repeated-measures analysis of variance or the Friedman test, as appropriate, based on the normality of data distribution. *Post hoc* analyses with correction for multiple comparisons were applied when necessary. For qualitative variables, differences between timepoints were evaluated using Cochran’s Q test.

Correlations between changes in CT and the CRP level and variations in other parameters were assessed using Pearson’s correlation coefficient for normally distributed variables and Spearman’s rank correlation coefficient for non-normally distributed variables. Additionally, Bland–Altman plots were generated to evaluate the agreement between paired measurements over time.

Improvement in various parameters was compared between patients with and without rotator-cuff disease. Differences in qualitative variables were analyzed using Fisher’s exact test, whereas quantitative variables were compared using the Mann–Whitney U test.

Statistical analyses were performed using IBM SPSS Statistics version 27.0 (IBM Corp., Armonk, NY, United States). Statistical significance was set at two-sided *p* < 0.05.

## 3 Results

### 3.1 Demographic and clinical characteristics

This study included 20 patients diagnosed with PMR between November 2023 and April 2025. The mean age was 73.5 ± 11.3 years, and 12 patients (60%) were female. The mean baseline CT was 242.1 ± 79 μm, and the mean disease duration until diagnosis was 12 ± 7 weeks. Other baseline characteristics are represented in Tables 1, 2. One patient did not report for the 6-month follow-up.

**Table 1:** Baseline characteristics.

Variable	Mean ± SD or Median-IQR
Prednisone dose	15 mg (IQR, 13.1–18.7)
CRP	1.47 mg/dL (IQR, 0.93–2.19)
ESR	43.5 ± 33.5 mm/h
VAS pain	8.5 (IQR, 8–9.75)
PGA	8 (IQR, 7–8)
MST	74.5 ± 46.7 min
PMR-AS	27.22 ± 7.34
PMR-ASimp	27.7 (IQR, 23.8–35)

CRP=C-reactive protein; ESR, erythrocyte sedimentation rate; VAS, visual analog scale; PGA, physician’s global assessment; MST, morning stiffness; PMR-AS, PMR disease activity score; PMR-ASimp, imputed PMR-AS.

**Table 2:** Baseline elevation upper limbs (EUL) grades and ultrasound (US) findings.

Variable	Patients (%)
EUL grades	
Grade 0	2 (10.5%)
Grade 1	3 (15.7%)
Grade 2	13 (65%)
Grade 3	2 (10.5%)
US findings	
BT	16 (84.2%)
SASD bursitis	9 (47.4%)
Trochanteric bursitis	16 (84.2%)
Hip sinovitis	8 (42%)
Comorbidities	
Dyslipidemia	8 (42%)
Hypertension	7 (36.8%)
Diabetes Mellitus	2 (10.5%)
Heart Disease	2 (10.5%)
Hypothyroidism	1 (5.2%)

EUL, elevate the upper limbs; US, ultrasound; BT, biceps tenosynovitis; SASD, subacromial and subdeltoid.

CT decreased significantly after 3 months (229.9 ± 79 μm, *p* = 0.017) and after 6 months (220.4 ± 76 μm, *p* = 0.014). Similarly, the CRP level, prednisone dose, VAS score for pain, PGA score, MST, PMR-AS, and PMR-ASimp decreased significantly after 3 months (*p* < 0.001, *p* = 0.0035, *p* < 0.001, *p* = 0.0015, *p* = 0.0006, *p* = 0.0002 and *p* < 0.0001, respectively) and after 6 months (*p* = 0.0004, *p* < 0.0001, *p* < 0.001, *p* < 0.0001, *p* < 0.0001, *p* < 0.0001 and *p* < 0.0001, respectively). Comparison of MSK US findings between baseline and 6 months revealed a significant decrease in BT (*p* = 0.039) (Supplementary Figure S1).

### 3.2 Correlations

No correlation was found between CT and the other inflammatory parameters at any time of follow-up (Supplementary Table S1). A significant correlation was found between CRP and a prednisone dose at 3 months after treatment (*p* = 0.002), but not at baseline or at 6 months (*p* = 0.659 and 0.241, respectively) (Supplementary Table S2). However, we found no correlation between CRP and the other parameters.

There was no correlation between changes in CT and the other parameters (Supplementary Table S3). Changes in CRP were correlated with changes in ESR of 12 patients (determination of ESR available in all visits), from baseline to three and 6 months and from three to 6 months, was statistically significant (*p* = 0.015, *p* = 0.018 and *p* = 0.006, respectively) (Supplementary Table S4).

### 3.3 Concordances

**Figure 3:**
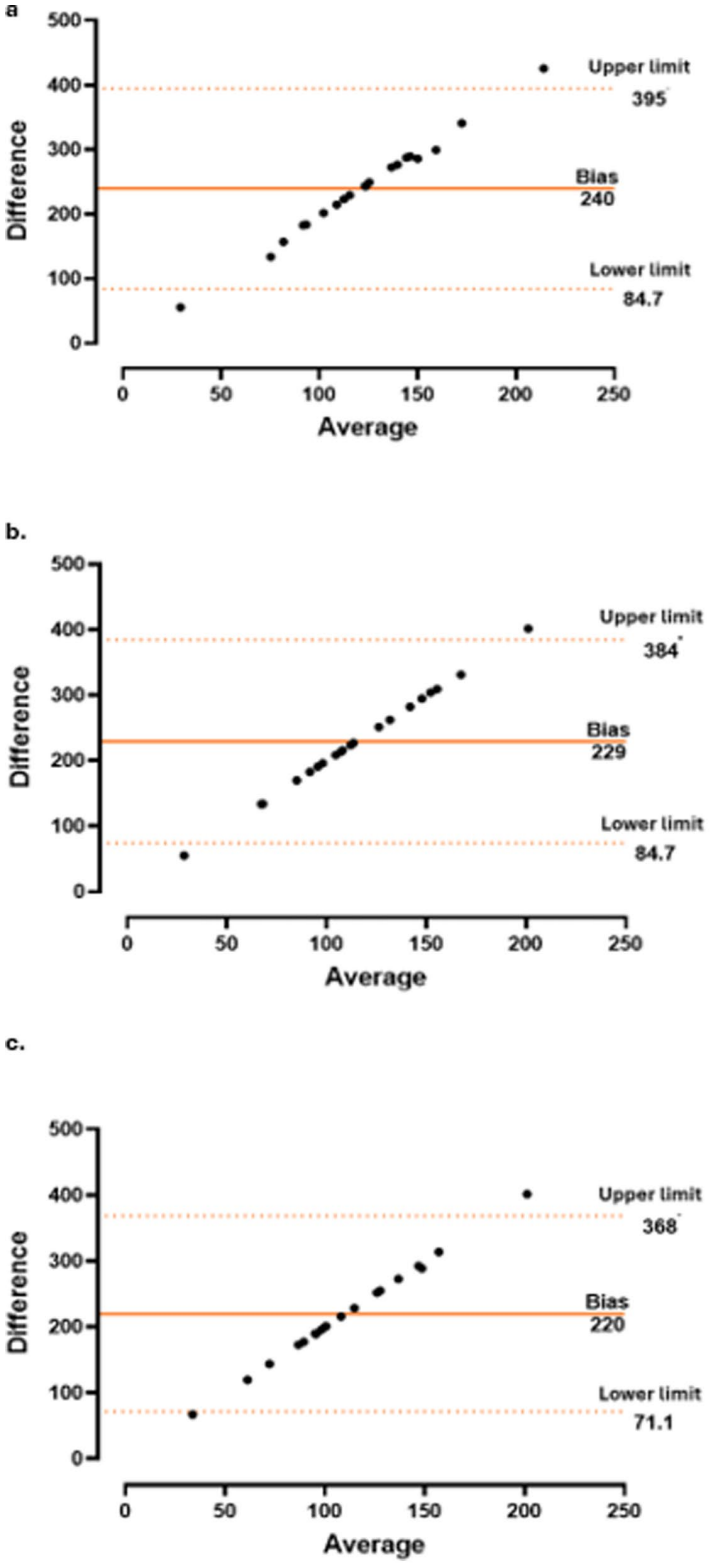
Concordance between choroidal thickness (CT) and c-reactive protein (CRP) at baseline **(a)**, 3 months **(b)** and 6 months **(c)**.

**Figure 4:**
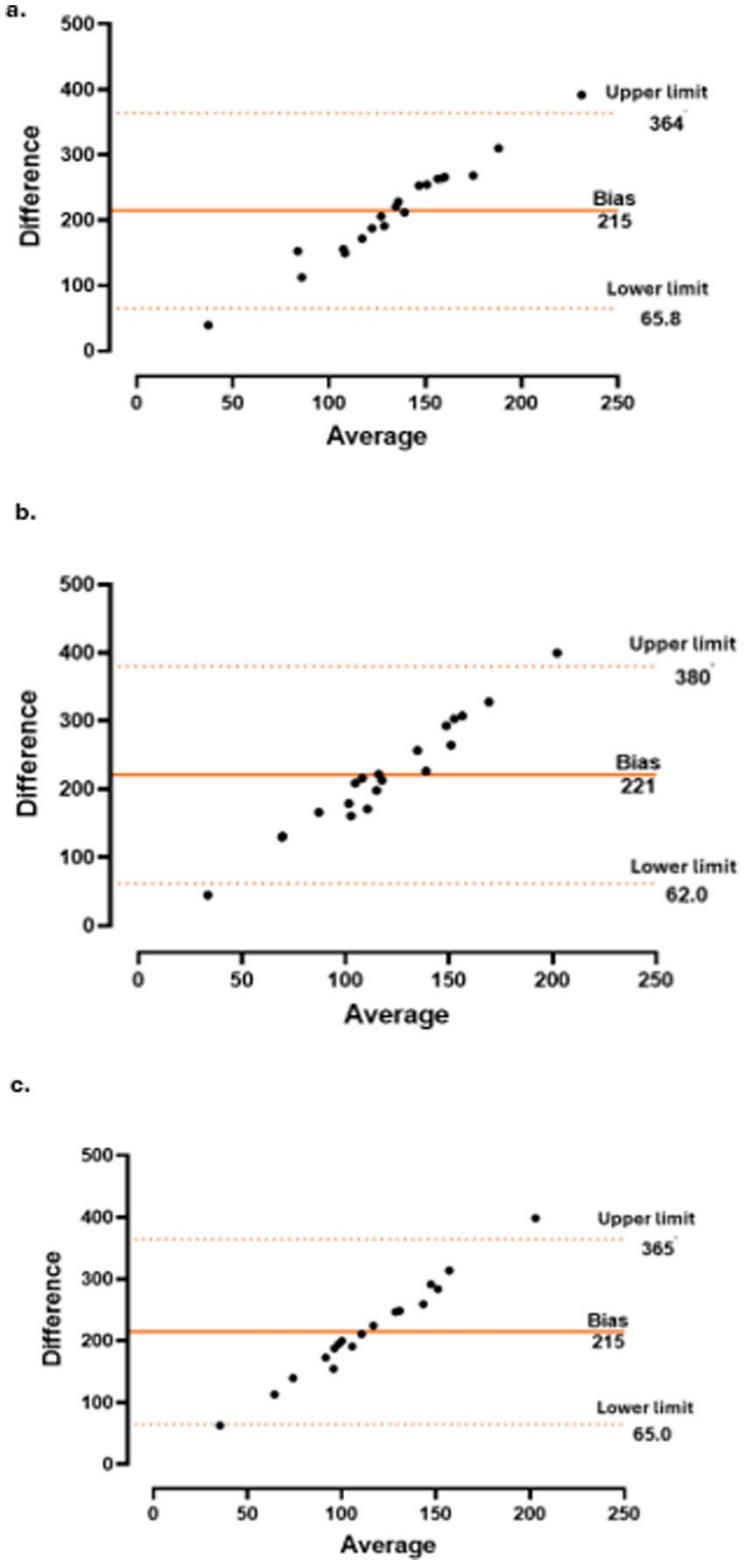
Concordance between choroidal thickness (CT) and polymyalgia rheumatica disease activity score (PMR-AS) at baseline **(a)**, 3 months **(b)** and 6 months **(c)**.

**Figure 5:**
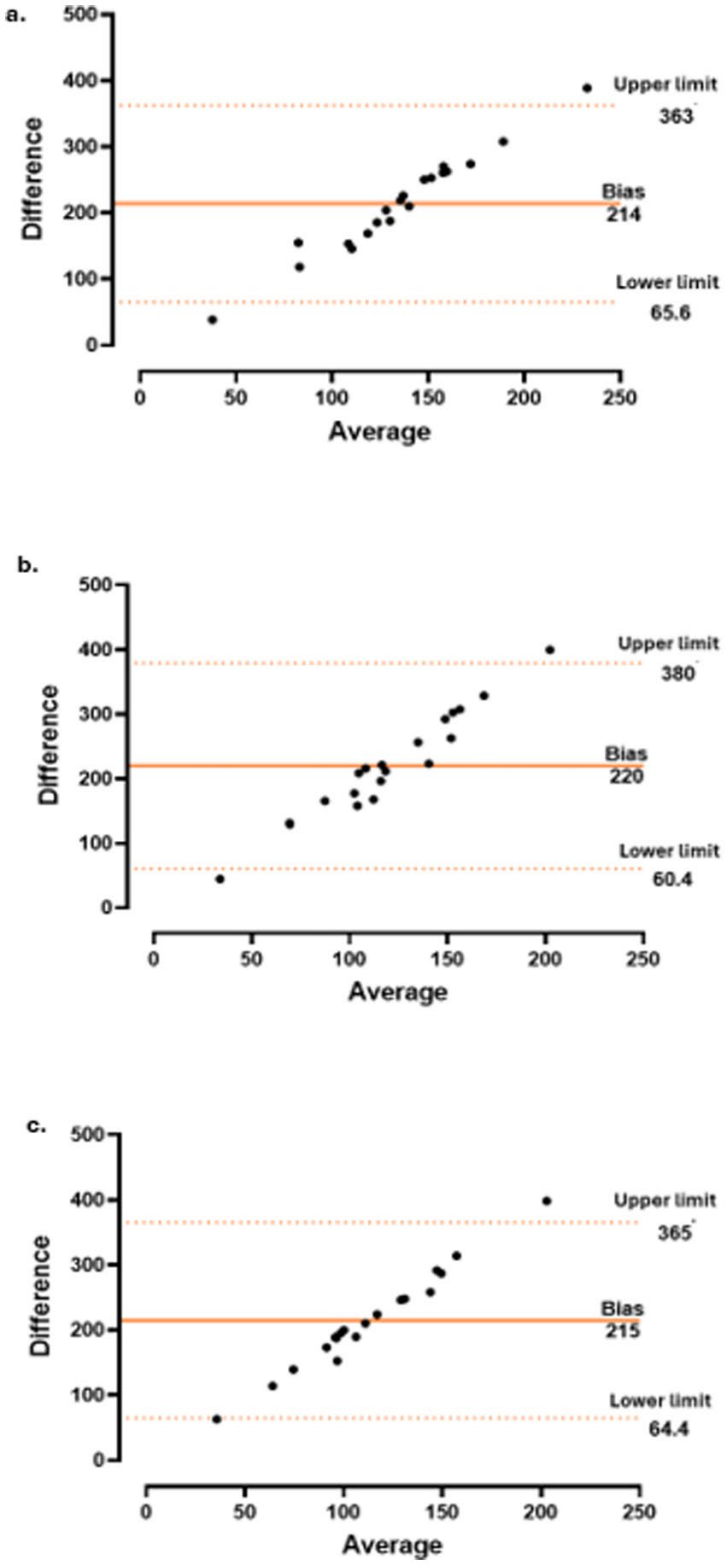
Concordance between choroidal thickness (CT) and imputed polymyalgia rheumatica disease activity score (PMR-ASimp) at baseline **(a)**, 3 months **(b)** and 6 months **(c)**.

The concordance between CT and CRP level (Figure 3), PMR-AS (Figure 4), and PMR-ASimp (Figure 5) at baseline and after 3 and 6 months of treatment was 95%.

### 3.4 Rotator cuff pathology

Of the 16 (100%) patients with BT on MSK US at baseline, seven (43.8%) had rotator-cuff pathology. BT evolution at 3 and 6 months was not significantly different among patients with and without rotator-cuff pathology (*p* = 0.650 and *p* = 0.057, respectively). Furthermore, changes in the VAS scores for pain at 3 and 6 months were not significantly different between patients with and without rotator-cuff involvement (*p* = 0.858 and *p* = 0,408 respectively).

## 4 Discussion

To the best of our knowledge, this is the first study to demonstrate that CT decreases significantly in patients with active PMR after 3 and 6 months of corticosteroid therapy. Furthermore, the 95% concordance between CT and CRP level, PMR-AS, and PMR-ASimp suggests that CT is a useful non-invasive biomarker of systemic inflammation in patients with PMR.

Previous studies evaluated the platelet-to-lymphocyte and neutrophil-to-lymphocyte ratios and their correlations with APR as possible predictors of evolution and treatment response in patients with PMR; however, no threshold has been determined, and the usefulness of these variables for monitoring inflammatory activity is controversial (30, 31).

Van der Geest et al. compared the serum levels of 26 biomarkers in patients with PMR to those in healthy individuals and reported that interleukin-6 (IL-6) and B-cell-activating factor (BAFF) levels were elevated in most patients with PMR at diagnosis and decreased significantly after 3 months of corticosteroid therapy. Moreover, APR levels were strongly correlated with PMR (32). Nevertheless, evidence for the use of these biomarkers in daily clinical practice is scarce (33). Furthermore, tests for some biomarkers are not available in all clinical centres, because of their high price or technical difficulties. In this study, the CRP level was most frequently used to determine PMR activity. Although no significant correlation was observed between CRP levels and ESR at baseline and at the 3- and 6-month follow-ups, a significant correlation between changes in CRP and ERS was observed at all timepoints. This suggests that any APR is useful for monitoring inflammatory activity over time despite the lack of consistent agreement at specific timepoints.

Biomarkers of systemic inflammation are important to evaluate treatment outcomes in daily practice. APR, such as CRP or ESR, are inflammatory biomarkers included in the PMR classification criteria. Although both markers allow clinicians to identify a state of systemic inflammation and its evolution after treatment, CRP is more sensitive (5, 34). Nevertheless, few observational studies have reported that 1.1–22.5% of patients have normal APR levels at diagnosis making monitoring of inflammatory activity more difficult in these patients (35, 36). CT measurement is particularly useful in patients with PMR with low or normal baseline CRP levels. Additionally, infectious diseases can increase CRP levels, leading to misinterpretation of the results.

In this study, the concordance between CT and CRP, PMR-AS, and PMR-ASimp was 95%. A similar level of concordance between CT and CRP, both at baseline and after treatment, has been reported in patients with spondyloarthritis, supporting the potential of CT as a biomarker associated with treatment response. These patients had active spondyloarthritis with low CRP levels (23). Devauchelle et al. (29) assessed the correlation between three PMR activity scores: CRP-PMR-AS, clinical-PMR-AS (clin-PMR-AS), and imputed-PMR-AS (imp-PMR-AS). In clin-PMR-AS, CRP is excluded, whereas in imp-PMR-AS, CRP is replaced by a value imputed based on the other score items. They observed a significant correlation between CRP-PMR-AS and clin-PMR-AS, as well as between CRP-PMR-AS and imp-PMR-AS, with the latter demonstrating a stronger association. Additionally, the agreement among these scores was studied by D’Agostino et al. in patients treated with tocilizumab, both at baseline and after treatment. They also found good concordance among the three activity scores (7).

Although data on the correlation among different PMR activity indices are available in the literature, we consider that a reliable assessment of the inflammatory status of the disease should rely on the availability of objective measurements in addition to activity scales and physical examination. In this context, there is a clear need to identify and validate new biomarkers. Moreover, although other proposed biomarkers such as IL-6 and BAFF have also been studied as potential indicators of PMR activity, it is important to note, as indicated by van der Geest et al. (32) that while both are associated with disease activity PMR, they have limitations in specificity. Although IL-6 correlates well with inflammatory activity in PMR, its elevation is not exclusive to the disease. Similarly, BAFF is elevated in various autoimmune disorders, limiting its ability to differentiate PMR from other inflammatory diseases. Moreover, measuring IL-6 or BAFF levels is not always practical, as not all laboratories have the necessary assays and turnaround times are often longer. Thus, we believe that the 95% concordance of our proposed biomarker may represent a superior diagnostic option in terms of specificity and accuracy for PMR.

Therefore, in cases when APR data are not available or are potentially influenced by other circumstances, CT is a valid alternative to evaluate inflammatory activity in patients with PMR.

Currently, most ophthalmology practices have an OCT machine, and OCT can easily be used in daily practice to monitor systemic inflammation. Moreover, CT measurement is easy to perform and is not time-consuming. Previous studies on systemic inflammatory diseases affecting younger people, such as spondylarthritis, have evaluated CT changes (23). However, PMR affects older people in whom a coexistence of multiple comorbidities is frequent. Therefore, CT variation due to physiological and pathological situations should be considered during study designing to avoid misinterpretations. CT varies with age and circadian rhythm. Several studies on age-related choroidal thinning have reported a decrease of 15.6–14 *μ*m for each decade of life (37, 38). Other studies reported a decrease only after the age of 60 years (39). In a study evaluating CT in healthy individuals from different age groups, Wakatsuki et al. found that the mean CT in participants aged 60–80 years was 179 ± 48.8 μ (40). Moreover, in a study on diurnal variations in CT, Tan et al. observed a progressive decrease from 9 a.m. till 5 p.m., and similar results were reported in a Japanese study (41, 42). In this study, all CT measurements were performed between 9 a.m. and 1 p.m. Patients with hypertension or hypercholesterolemia have thicker choroids (43, 44). However, reports on the effect of diabetes mellitus on CT are contradictory (45). In summary, several conditions may influence CT. Therefore, our study design included individual follow-up of every patient, to avoid a variability in CT measurements at baseline due to comorbidities.

We did not find a strong correlation between CT or CRP level and disease activity indices and scales currently used in clinical settings at baseline or at the 3- and 6-month follow-ups. Although the CRP level was significantly correlated with the prednisone dose at 3 months, not significant correlation was observed at baseline or at 6 months after treatment. These findings suggest that at baseline and 6 months after treatment, the prednisone dose was adjusted independently of CRP levels, likely based on clinical assessment or other inflammatory parameters. In contrast, at the 3-month follow-up, CRP levels likely guided prednisone dose adjustments.

In this study, changes in CT or CRP levels did not show significant correlations with disease activity indices or scales at any time point. While these findings may partly reflect the limited sample size, they also highlight important considerations regarding the complex relationship between objective biomarkers and clinical disease activity. Previous work by Steiner et al. (23) similarly failed to demonstrate a correlation between CT levels and activity scores in spondylarthritis, suggesting that this discordance is not merely attributable to underpowered analyses. Their interpretation, which attributes this discordance to the influence of highly subjective parameters, highlights the inherent variability of patient-reported outcomes and underscores the need for more robust and objective measures of disease activity. Furthermore, the measurement ranges of CT (typically 250–500 μm) and CRP levels (0 to >100 mg/dL) differ substantially from the scoring ranges of patient- or physician-assessed indices (0–10 points). Such discrepancies in scale metrics may also contribute to the observed discordance, as proportional changes across these measures are not directly comparable.

In addition, as Yuvaci et al. (46) concluded, given the absence of a correlation between CT and ESR, APR should be regarded as nonspecific systemic markers of inflammation. Their levels may be influenced by age, sex and comorbidities, and therefore may not accurately reflect PMR-related inflammation at a given timepoint. Moreover, systemic inflammation may manifest in a heterogeneous manner, such that it does not affect all tissues uniformly. Also, even under a successful treatment regimen, mild subclinical inflammationmay persist (47). So, CT may serve as a complementary tool for assessing inflammatory activity, but further studies are needed to validate its role as an independent biomarker (48).

MSK US findings are included in the PMR classification criteria, because several studies have demonstrated the usefulness of this technique for detecting inflammatory lesions in patients with PMR. The most frequently reported MSK US abnormality in patients with PMR is SASD bursitis followed by BT (49). However, in this study, BT was more frequently observed. In patients with PMR, concurrent degenerative rotator-cuff pathology may occur, leading to BT or SASD bursitis (50, 51). In such patients, evaluating whether persistent BT or SASD bursitis after treatment is caused by persistent inflammatory activity or mechanical pathology is difficult. Few longitudinal studies have evaluated decrease in inflammatory images on MSK US after treatment. Macchioni et al. reported improvement of inflammatory lesions in the shoulders on MSK US after 6 months of corticosteroid therapy; however, in few patients inflammation persisted despite clinical improvement (52). Other studies reported no correlation between MSK US findings and clinical parameters in PMR (53, 54). In this study, evolution of BT findings was not significantly different between patients with or without rotator-cuff pathology. Our results suggest that mechanical shoulder involvement does not influence evolution of BT. Furthermore, changes in the VAS scores for pain were not significantly different between patients with and without rotator-cuff pathology. These findings are consistent with the notion that pain in PMR may be primarily related to inflammatory disease. Nevertheless, additional research with larger sample sizes are warranted to explore this relationship in greater depth.

### 4.1 Study limitations

This study had some limitations. The main limitation of the study is the small simple size which may markedly compromise the study’s statistical power and impede the extent to which its findings can be generalized to the broader population of patients with PMR. Another limitation is the lack of a control group. Ideally, comparisons should be made with individuals of similar age. In diseases affecting younger individuals, where significant comorbidities are unlikely—such as axial spondyloarthritis—CT changes can be compared with a healthy control group. However, in conditions affecting older individuals, comparisons with a control group are subject to multiple confounding factors. Therefore, given the variability in age at PMR onset, the presence of pre-existing comorbidities, and the potential effects of treatments, we consider that the most appropriate approach is to monitor CT changes in each patient individually. Designing a homogeneous patient cohort is unfeasible, and identifying a truly healthy control group is even more challenging, as multiple potentially undiagnosed conditions that could influence CT are common in this age group. Future research should incorporate strategies to better account for these variables to provide a more accurate assessment of CT changes over time. Reliability bias was avoided because MSK US examination was performed by the same clinician. The use of different devices in the centres could be another shortcoming; however, previous studies have reported that subfoveal CT measurements from different devices may be used interchangeably with a high degree of consistency (55, 56). ESR values were not available for all patients throughout the 6-month study period. Consequently, although correlations between ESR and CT or CRP were assessed, these findings should be interpreted with caution, as incomplete data may have introduced bias. We did not examine subclinical arterial lesions, as this was not an objective of the present study and because we did not suspect an association with GCA in our patients. All included patients demonstrated a favorable clinical course and response to treatment. We acknowledge that future research with a larger patient cohort will be necessary to confirm our findings, including an evaluation of subclinical arterial lesions, especially in patients with a refractory clinical course or corticosteroid resistance.

## 5 Conclusion

In conclusion, this pilot study demonstrated that CT is high in patients with recently diagnosed PMR and decreases significantly after 3 and 6 months of corticosteroid therapy. The 95% concordance between CT and CRP level and PMR activity scores suggests that CT is a useful inflammatory biomarker in patients with PMR. As a pilot study, our results are preliminary; therefore, future research with larger cohorts is recommended to enhance statistical power and validate the proposed hypothesis.

## Data Availability

The original contributions presented in the study are included in the article/supplementary material, further inquiries can be directed to the corresponding author/s.
