# Supplementary material for: Choroidal thickness as a biomarker of systemic inflammation in patients with polymyalgia rheumatica
Source: Front Med (Lausanne). 2025 Oct 17;12:1689327. doi: 10.3389/fmed.2025.1689327 (PMC12575362; doi:10.3389/fmed.2025.1689327)
Supplement: Supplementary file 1 [file Data_Sheet_1.docx]

Supplementary Material

**Figure S1. Evolution from baseline to 3 months; baseline to 6 months and 3 months to 6 months visits. Choroidal thickness (CT) (a); c-reactive protein (CRP) (b); prednisone dose (c); visual analogue scale of pain´s patient (VAS pain) (d); physician global assessment (PGA) (e); morning stiffnes (MST) (f); PMR disease activity index (PMR-AS) (g); imputed PMR-AS (h) and bicipital tenosynovitis (BT) (i)**


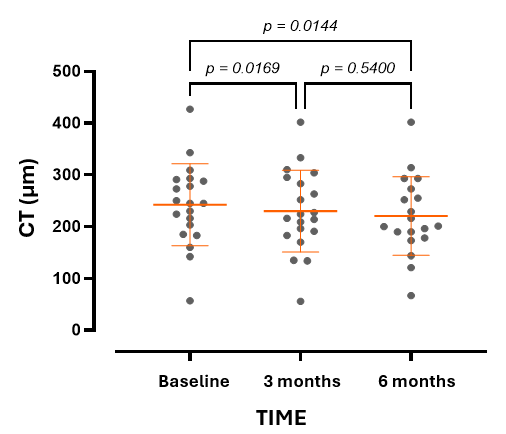

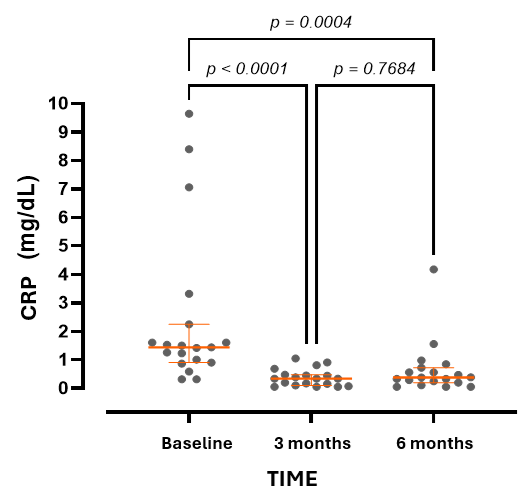


**d.**

**c.**

**b.**

**a.**


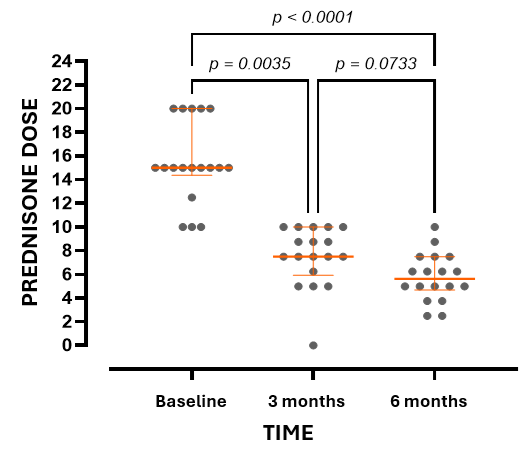

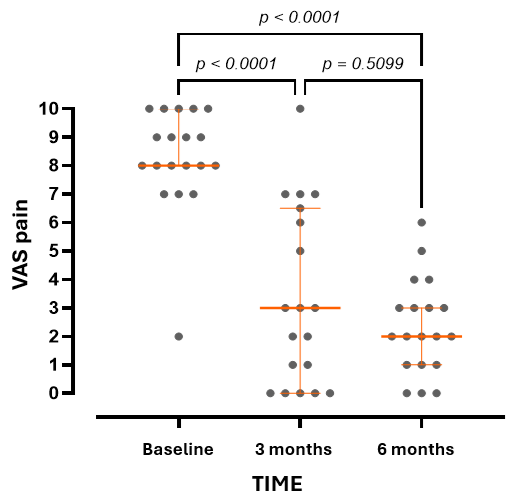


**f.**

**e.**


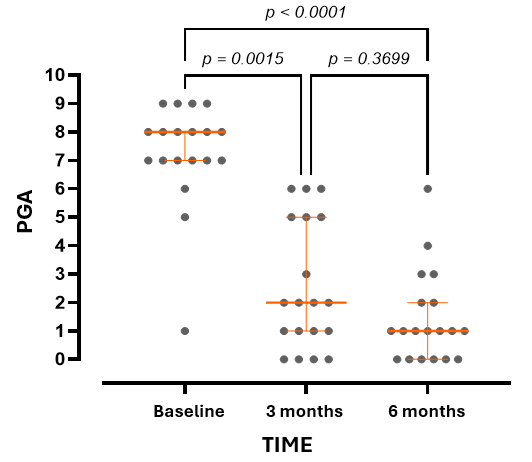

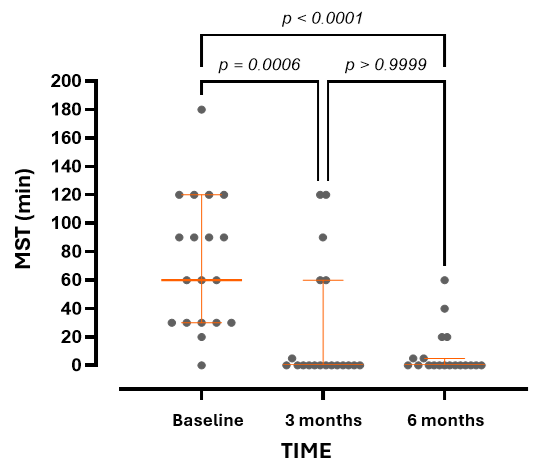


**g.**

**h.**


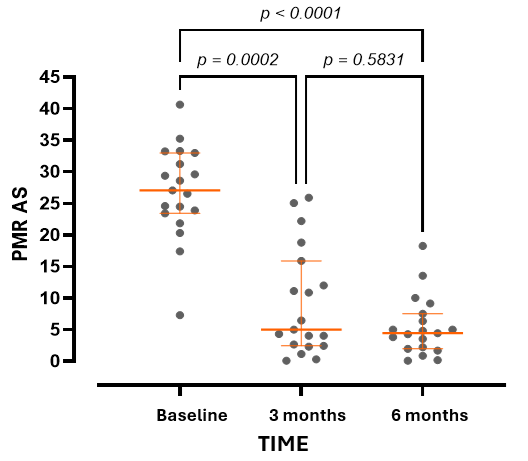

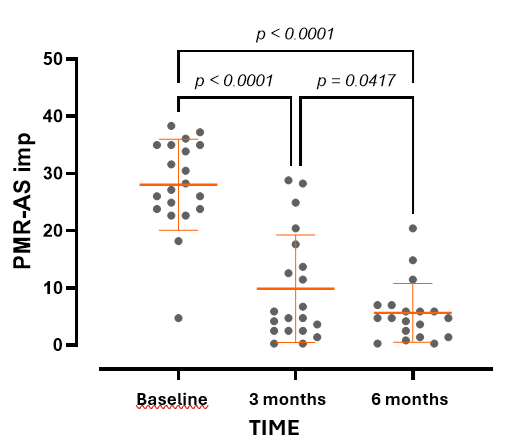


**i.**


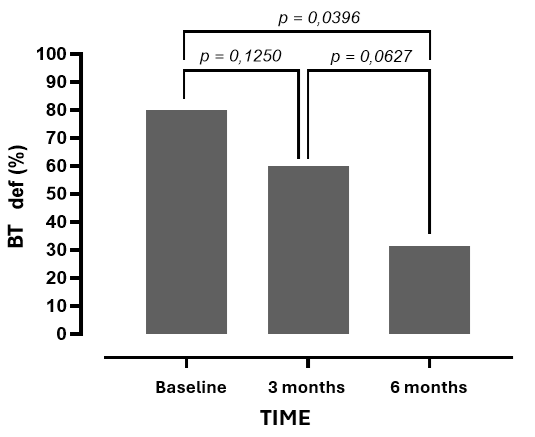


**Table S1. Correlation between CT and the other inflammatory parameters in each visit**

| T  **Variable** | **r** | IC  **IQR** | **p-value** |
| --- | --- | --- | --- |
| **Baseline** |  |  |  |
| CRP | 0.033 | -0.427-0.479 | 0.889 |
| Dose of prednisone | -0.138 | -0.557-0.336 | 0.560 |
| ESR | 0.153 | -0.351-0.588 | 0.544 |
| VAS pain | -0.235 | -0.622-0.245 | 0.319 |
| PGA | -0.422 | -0.735-0.039 | 0.063 |
| MST | 0.423 | -0.038-0.736 | 0.063 |
| PMR-AS | -0.210 | -0.606-0.269 | 0.374 |
| PMR-Asimp | -0.159 | -0.572-0.317 | 0.501 |
|  |  |  |  |
| **3 months** |  |  |  |
| CRP | 0.222 | -0.258-0.614 | 0.348 |
| Dose of prednisone | 0.313 | -0.164-0.671 | 0.179 |
| ESR | -0.210 | -0.637-0.315 | 0.415 |
| VAS pain | -0.393 | -0.718-0.074 | 0.087 |
| PGA | -0.286 | -0.655-0.192 | 0.221 |
| MST | 0.048 | -0.415-0.491 | 0.842 |
| PMR-AS | -0.231 | -0.619-0.249 | 0.327 |
| PMR-Asimp | -0.283 | -0.653-0.196 | 0.226 |
|  |  |  |  |
| **6 months** |  |  |  |
| CRP | 0.023 | -0.447-0.484 | 0.923 |
| Dose of prednisone | -0.100 | -0.552-0.398 | 0.693 |
| ESR | -0.182 | -0.619-0.341 | 0.481 |
| VAS pain | -0.084 | -0.529-0.397 | 0.731 |
| PGA | -0.184 | -0.598-0.306 | 0.452 |
| MST | -0.151 | -0.576-0.338 | 0.536 |
| PMR-AS | -0.078 | -0.525-0.402 | 0.750 |
| PMR-Asimp | -0.109 | -0.546-0.376 | 0.658 |

*CT=* *choroidal thickness;* *CRP=C-reactive protein; ESR=erythrocyte sedimentation rate; VAS= visual analogue scale; PGA= physician´s global assessment; MST= morning stiffness; PMR-AS= PMR disease activity score; PMR-ASimp= imputed PMR-AS*

**Table S2. Correlation between CRP and the other inflammatory parameters in each visit**

| T  **Variable** | **r** | IC  **IQR** | **p-value** |
| --- | --- | --- | --- |
| **Baseline** |  |  |  |
| CT | 0.033 | -0.427-0.479 | 0.889 |
| Dose of prednisone | 0.105 | -0.366-0.533 | 0.659 |
| ESR | 0.465 | -0.017-0.772 | 0.052 |
| VAS pain | -0.221 | -0.613-0.245 | 0.345 |
| PGA | -0.057 | -0.409-0.498 | 0.809 |
| MST | -0.051 | -0.494-0.412 | 0.828 |
| PMR-AS | 0.208 | -0.271-0.605 | 0.378 |
| PMR-Asimp | -0.096 | -0.527-0.373 | 0.684 |
|  |  |  |  |
| **3 months** |  |  |  |
| CT | 0.222 | -0.258-0.614 | 0.348 |
| Dose of prednisone | 0.655 | 0.286-0.855 | **0.002** |
| ESR | 0.313 | -0.212-0.698 | 0.220 |
| VAS pain | -0.194 | -0.595-0.285 | 0.413 |
| PGA | 0.014 | -0.443-0.464 | 0.954 |
| MST | 0.033 | -0.426-0.480 | 0.888 |
| PMR-AS | 0.029 | -0.430-0.477 | 0.902 |
| PMR-Asimp | -0.071 | -0.509-0.395 | 0.763 |
|  |  |  |  |
| **6 months** |  |  |  |
| CT | 0.023 | -0.447-0.484 | 0.923 |
| Dose of prednisone | 0.291 | -0.218-0.676 | 0.241 |
| ESR | 0.066 | -0.441-0.541 | 0.800 |
| VAS pain | 0.095 | -0.388-0.536 | 0.699 |
| PGA | -0.241 | -0.635-0.252 | 0.319 |
| MST | -0.327 | -0.688-0.163 | 0.172 |
| PMR-AS | 0.065 | -0.413-0.515 | 0.791 |
| PMR-Asimp | -0.005 | -0.469-0.461 | 0.983 |

*CT=choroidal thickness;* *CRP=C-reactive protein; ESR=erythrocyte sedimentation rate; VAS= visual analogue scale; PGA= physician´s global assessment; MST= morning stiffness; PMR-AS= PMR disease activity score; PMR-ASimp= imputed PMR-AS*

**Table S3. Correlation between changes in CT and the other inflammatory parameters**

| T  **Variable** | **r** | IC  **IQR** | **p-value** |
| --- | --- | --- | --- |
| **Baseline to 3 months** |  |  |  |
| CRP | -0.127 | -0.549-0.346 | 0.593 |
| Dose of prednisone | -0.211 | -0.601-0.268 | 0.372 |
| ESR | 0.149 | -0.408-0.624 | 0.595 |
| VAS pain | -0.296 | -0.661-0.182 | 0.204 |
| PGA | -0.422 | -0.735-0.039 | 0.063 |
| MST | -0.038 | -0.483-0.423 | 0.874 |
| PMR-AS | -0.209 | -0.606-0.269 | 0.374 |
| PMR-Asimp | -0.159 | -0.572-0.316 | 0.501 |
|  |  |  |  |
| **Baseline to 6 months** |  |  |  |
| CRP | -0.264 | -0.649-0.230 | 0.276 |
| Dose of prednisone | -0.145 | -0.583-0.358 | 0.566 |
| ESR | -0.078 | -0.564-0.447 | 0.772 |
| VAS pain | 0.012 | -0.456-0.475 | 0.959 |
| PGA | -0.288 | -0.665-0.205 | 0.231 |
| MST | 0.119 | -0.367-0.554 | 0.627 |
| PMR-AS | 0.019 | -0.450-0.480 | 0.937 |
| PMR-Asimp | 0.069 | -0.409-0.518 | 0.777 |
|  |  |  |  |
| **3 months to 6 months** |  |  |  |
| CRP | -0.452 | -0.758-0.016 | 0.052 |
| Dose of prednisone | -0.204 | -0.621-0.304 | 0.417 |
| ESR | -0.233 | -0.689-0.355 | 0.418 |
| VAS pain | -0.183 | -0.598-0.308 | 0.451 |
| PGA | -0.035 | -0.492-0.438 | 0.888 |
| MST | 0.121 | -0.365-0.555 | 0.621 |
| PMR-AS | -0.171 | -0.589-0.320 | 0.484 |
| PMR-Asimp | -0.164 | -0.585-0.326 | 0.501 |

*CT=choroidal thickness; CRP=C-reactive protein; ESR=erythrocyte sedimentation rate; VAS= visual analogue scale; PGA= physician´s global assessment; MST= morning stiffness; PMR-AS= PMR disease activity score; PMR-ASimp= imputed PMR-AS*

**Table S4. Correlation between changes of CRP and the other inflammatory parameters**

| T  **Variable** | **r** | IC  **IQR** | **p-value** |
| --- | --- | --- | --- |
| **Baseline to 3 months** |  |  |  |
| CT | -0.127 | -0.549-0.346 | 0.593 |
| Dose of prednisone | 0.139 | -0.335-0.557 | 0.558 |
| ESR | 0.625 | 0.149-0.865 | **0.015** |
| VAS pain | 0.174 | -0.303-0.582 | 0.462 |
| PGA | 0.229 | -0.250-0.619 | 0.330 |
| MST | -0.047 | -0.490-0.416 | 0.844 |
| PMR-AS | 0.282 | -0.197-0.652 | 0.228 |
| PMR-Asimp | 0.006 | -0.448-0.458 | 0.979 |
|  |  |  |  |
| **Baseline to 6 months** |  |  |  |
| CRP | -0.264 | -0.649-0.230 | 0.276 |
| Dose of prednisone | 0.234 | -0.275-0.640 | 0.351 |
| ESR | 0.588 | 0.115-0.844 | **0.018** |
| VAS pain | -0.117 | -0.553-0.368 | 0.632 |
| PGA | 0.291 | -0.203-0.661 | 0.227 |
| MST | -0.043 | -0.498-0.432 | 0.862 |
| PMR-AS | 0.168 | -0.322-0.587 | 0.491 |
| PMR-Asimp | -0.041 | -0.497-0.432 | 0.866 |
|  |  |  |  |
| **3 months to 6 months** |  |  |  |
| CRP | -0.452 | -0.758-0.016 | 0.052 |
| Dose of prednisone | -0.049 | -0.515-0.439 | 0.846 |
| ESR | 0.700 | 0.254-0.900 | **0.006** |
| VAS pain | 0.360 | -0.127-0.707 | 0.129 |
| PGA | 0.267 | -0.233-0.648 | 0.281 |
| MST | 0.055 | -0.421-0.508 | 0.821 |
| PMR-AS | 0.442 | -0.030-0.753 | 0.058 |
| PMR-Asimp | 0.428 | -0.046-0.745 | 0.067 |

*CT=choroidal thickness;* *CRP=C-reactive protein; ESR=erythrocyte sedimentation rate; VAS= visual analogue scale; PGA= physician´s global assessment; MST= morning stiffness; PMR-AS= PMR disease activity score; PMR-ASimp= imputed PMR-AS*
